# Interaction of urea with frequency and amount of distillers grains supplementation for growing steers on a high forage diet

**DOI:** 10.1093/tas/txac076

**Published:** 2022-06-05

**Authors:** Haley F Linder, Josh E Sebade, Zac E Carlson, Hannah C Wilson, Tyler J Spore, Mary E Drewnoski, Jim C MacDonald

**Affiliations:** Department of Animal Science, University of Nebraska–Lincoln, NE 68583, USA; Department of Animal Science, University of Nebraska–Lincoln, NE 68583, USA; Department of Animal Science, University of Nebraska–Lincoln, NE 68583, USA; Department of Animal Science, University of Nebraska–Lincoln, NE 68583, USA; Department of Animal Science, University of Nebraska–Lincoln, NE 68583, USA; Department of Animal Science, University of Nebraska–Lincoln, NE 68583, USA; Department of Animal Science, University of Nebraska–Lincoln, NE 68583, USA

**Keywords:** beef cattle, distillers grains plus solubles, supplementation frequency, urea

## Abstract

Two studies were conducted to determine interactions of urea inclusion to a dried distillers grains plus solubles (DDGS; 29.4% crude protein, 5.48% ether extract) supplement fed at two amounts and two frequencies to steers on a high forage diet. In Exp. 1, 120 (247 kg; SD = 20) steers were fed individually for 84 d. Steers received ad libitum grass hay (6.8% crude protein) and one of eight treatments. Treatment design was a 2 × 2 × 2 factorial. Supplement was fed daily or three times per week, amount of supplement fed was 6.36 kg dry matter (DM)/week [0.37% body weight (BW); LO] or 12.73 kg DM/week (0.74% BW; HI) and contained either no urea or 1.3% urea on a DM basis. Steer BW was measured at the start and end of the trial and hay DM intake (DMI) was measured weekly. In Exp. 2, ruminally cannulated steers (310 kg; SD = 25) were used in a row-column design with eight steers and six 14-d periods. Treatments assigned were the same as Exp. 1, except that supplement was fed at 0.4% of BW (LO) or 0.8% of BW (HI) and supplement was fed either daily (DY) or every other day (ALT). Hay DMI, rumen ammonia-N, rumen pH, in situ neutral detergent fiber (NDF) disappearance, and rumination were measured. In Exp. 1, average daily gain (ADG) was affected by amount of supplement with steers on HI gaining 0.30 kg/d more (*P* < 0.01) than LO. Hay DMI was reduced by increased amount of supplement (0.39 kg/d; *P* < 0.01) and by decreased frequency of supplementation (0.54 kg/d; *P* < 0.01). In Exp. 2, hay DMI was also reduced due to increased amount of supplement and decreased frequency of supplementation (*P* < 0.01). Rumen pH was decreased on the day of supplement feeding for steers on ALT (*P* < 0.01) and reduced for steers fed HI vs. LO. There was no difference in NDF digestibility between DY and ALT (*P* > 0.05). For ALT steers, there was reduction (*P* < 0.01) in in situ NDF disappearance for the HI compared to LO amount of supplementation on the day of supplementation. Infrequent supplementation of DDGS results in no difference in ADG but decreased hay DMI compared to daily supplementation. Urea had no effect on digestion or ADG, suggesting rumen degradable protein was not deficient when supplementing DDGS. There is little change in rumen fermentation parameters between frequency of supplement feeding, indicating that forage digestion is not impacted by supplementation frequency. Dried distillers grains can be supplemented infrequently without a reduction in animal performance.

## INTRODUCTION

Reducing supplementation frequency is a strategy to reduce labor costs in backgrounding cattle operations. In the state of Nebraska, a popular supplement for growing cattle consuming forage-based diets is dried distillers grains (DDGS), due to its cost and nutrient content. Unlike traditional energy supplements that provide energy in the form of non-structural carbohydrates (NSC), distillers grains provides energy in the form of highly digestible fiber and rumen undegradable protein (RUP), which may reduce negative associative effects observed with NSC supplementation, such as decreased digestibility of the forage component of the diet (Caton and Dhuyvetter, 1997). However, reducing supplementation frequency of DDGS from daily to alternate day may reduce average daily gain (ADG) by 10% (Loy et al., 2008; Stalker et al., 2009). However, not all infrequent supplementation strategies on forage-based diets have been reported to cause decreases in animal performance. When Drewnoski et al. (2011) fed a supplement of corn gluten feed (CGF) and soyhulls (SH), there was no reduction in growing steer performance from daily to 2×/week supplementation frequency. This supplement was similar to DDGS in terms of being highly digestible, but low in NSC; however, a key difference was that much of the crude protein (CP) content of CGF is rumen degradable protein (RDP), thus being readily available to contribute to the rumen available nitrogen (RAN) pool and microbial needs.

In the case of DDGS, the protein content is high in RUP but low in RDP. For cattle consuming a low-quality forage (<7% CP), RDP is often the first limiting nutrient (McCollum and Horn, 1990). Inadequate amounts of RDP can impair rumen microbial fermentation as fibrolytic bacteria are most sensitive to RAN (Köster et al., 1996). When a large amount of protein bypasses the rumen before microbes can access the nitrogen, forage digestion may be reduced and animal performance may be negatively impacted since the digestion of the forage, which makes up the largest portion of their diet, is not maximized. However, the ruminant animal is efficient in salvaging nitrogen to balance a RAN deficiency, especially in times of low dietary protein intake (Wickersham et al., 2008). When protein is fed above the animal’s metabolizable protein (MP) requirement, nitrogen (N) can be cleaved in the liver and recycled to the rumen in the form of urea thus, contributing to the RAN supply. In backgrounding operations, DDGS is often fed in excess of metabolizable protein requirements as excess protein can be used for energy. Stalker et al. (2007) determined that performance was not improved when urea, an RDP source, was added to the DDGS supplement fed to growing calves consuming meadow hay and concluded feeding a DDGS supplement daily that provided excess MP, as predicted by the NRC (1996) model, would provide enough N through recycling to meet microbial demands. Therefore, it has been assumed that there is not an RDP deficiency when growing calves receive DDGS supplement in excess of MP requirements. However, while the inclusion of urea to a DDGS supplement did not impact animal performance when fed daily, providing DDGS infrequently may not allow for recycled nitrogen to contribute to the RAN supply at the time of peak microbial fermentation. Consequentially, RDP may be deficient and forage digestibility reduced. Additionally, the amount or rate of supplementation could contribute to impacts of supplementation frequency on ruminal digestion. The RDP-to-total digestible nutrients (TDN) ratio of a diet has been identified as an important factor in forage digestion (Cochran et al., 1998). As the amount of TDN increases, microbial needs for N also increase. Therefore, inadequate RDP relative to TDN could result in reduced forage utilization (Bodine et al., 2001). Increasing the TDN of a diet through increased amount or rate of supplementation could further exacerbate the deficiency of recycled nitrogen during infrequent supplementation of DDGS.

We hypothesized that the addition of urea to a DDGS supplement would immediately contribute to RAN if the animals’ nitrogen recycling system could not match microbial demands due to an infrequent supplementation pattern. Supplying urea at the time of supplementation would reduce a potential RDP deficiency and thus improve forage digestibility and subsequent animal performance. The objective of these studies was to determine the interaction of the inclusion of urea with a dried distillers grains supplement fed at either a low or high amount, and either daily or on alternative days.

## MATERIALS AND METHODS

All animal-use procedures were reviewed and approved by the Institutional Animal Care and Use Committee (protocol 1785) at the University of Nebraska–Lincoln.

### Exp. 1: Performance Trial

One hundred twenty crossbred steers (247 kg; SD = 20) were fed one of eight treatments for 84 d to determine the effects of the inclusion of urea with the frequency and amount of distillers grains supplement on growing steer performance. There were two groups (blocks), of 60 steers, that were fed in the same barn: block one was conducted November through February, and block two was March through June. Animals were stratified by body weight within block and randomly assigned to treatment. Treatments were arranged in a 2 × 2 × 2 factorial design, with factors including frequency of supplement feeding, amount of supplement, and addition of urea to supplement. There was a total of 15 animals per treatment. To balance the number of observations for each treatment across the whole experiment, treatments where there were seven steers assigned to a treatment in block one, then had eight steers assigned to that treatment in block two, and vice versa.

Steers were individually fed in a Calan gate system (American Calan, Northwood, NH). All steers received ad libitum smooth bromegrass hay, and free choice mineral blocks (American Stockman Big Six; Compass Minerals; Overland Park, KS) containing 96% NaCl; 2,400 ppm Mn; 2,400 ppm Fe; 260 ppm Cu; 320 ppm Zn; 70 ppm I; and 40 ppm Cu. Supplement was dried distillers grains with solubles (DDGS) with 2.25% limestone and 2.5% molasses (Table 1). The smooth bromegrass hay (*Bromus inermis*.; 6.8% CP and 68.9% neutral detergent fiber; dry matter-basis) was ground to pass through a 127 mm screen (Mighty Giant model 2015; Jones Manufacturing, Beemer, NE). Supplement was fed either every day (DY) or on Monday, Wednesday, and Friday (3X). Amount of supplement fed was 6.36 kg dry matter (DM)/week (LO) or 12.73 kg DM/week (HI), split equally between feedings. Steers on the DY LO and DY HI treatments received 0.91 and 1.82 kg DM/d, respectively. Steers on the 3X LO and 3X HI received 2.12 and 4.24 kg DM, respectively, on each Monday, Wednesday, and Friday. The amount of supplement remained the same throughout the trial and was equal to 0.37 (LO) or 0.74% (HI) of initial BW on a dry matter basis. Supplement contained either no urea (−U) or 1.3% urea (+U; Table 1). To ensure total consumption of supplement and ad libitum hay intake, hay was not fed until five hours post-supplement feeding. Supplement was fed at 0600 h.

**Table 1.** Composition of supplements fed to steers in both the performance and digestion trials

	Composition, % of dry matter (DM)	
Ingredient	DDGS	DDGS+U
Dried distillers grains plus solubles	95.25	93.95
Molasses	2.50	2.50
Limestone	2.25	2.25
Urea	–	1.30
Nutrient Composition		
Dry Matter, %	92.1	92.3
Organic Matter, % DM	92.7	93.0
Crude Protein, % DM	29.4	32.4
Ether Extract, % DM	5.48	5.51

Weekly composites of hay and supplements were used to determine nutrient content. The hay was ground to pass through a 1-mm screen using a Wiley Mill grinder. Neutral detergent fiber (NDF) was analyzed according to Van Soest et al. (1991) with the addition of 0.5 g of sodium sulfite and CP was analyzed using a combustion-type N analyzer (FlashSmart N/Protein Analyzer CE Elantech, Inc. Lakewood, NJ; AOAC International, 1999; method 990.03). Ash content was estimated using a 600 °C muffle furnace for 6 h (AOAC International, 1999; method 4.1.10) and the estimate was subsequently used to calculate organic matter content.

To adjust for gut fill, steers were fed a common diet of 50% Sweet Bran (Cargill Corn Milling; Blair, NE) and 50% alfalfa hay at 2% of BW for five days at the beginning and end of the trial (Watson et al., 2013). Both initial and final body weight (BW) were recorded for the last three consecutive days of the limit-feeding period using a hydraulic squeeze chute with mounted load cells (Silencer, Moly Manufacturing Inc.; Lorraine, KS: scale readability ± 0.90 kg). On the last day of the starting limited-feeding period, steers were implanted with 36 mg zeranol (Ralgro; Merck Animal Health; Madison, NJ). Amount of hay offered was recorded daily and refusals were collected weekly. Weekly orts were dried with forced air at 60 °C for 48 h to measure DM (AOAC International, 1999; method 4.2.03).

Data were analyzed using the MIXED Procedure of SAS (SAS Inst. Inc., Cary, NC). Four animals were removed from the analysis, two due to death, one due to chronic illness, and the other was a bull. Animal served as the experimental unit. The models for initial BW, final BW, average daily gain (ADG), and hay DM intake were first analyzed with the interaction of block and treatment since the experiment occurred in two blocks of time (November to February and March to June). However, this interaction was not significant for any variable and was subsequently removed from the model. The final model included block, amount of supplementation, frequency of supplementation, inclusion of urea, and all factorial interactions (amount by frequency, amount by urea inclusion, frequency by urea inclusion, and amount by frequency by urea inclusion). There were no significant (*P* < 0.05) factorial interactions so only the main effects are reported.

### Exp. 2: Digestion Trial

Eight ruminally cannulated crossbred steers (310 kg; SD = 25) were used in an eight by six row-column design with eight steers and six periods to determine effects of inclusion of urea with the frequency and amount of distillers grain supplementation on rumen digestion parameters. Treatment design was a 2 × 2 × 2 factorial, with factors including amount of supplementation, frequency of supplementation, and inclusion of urea. Steers received supplement at 2.8% (LO) or 5.6% (HI) of BW per week. Supplement amount was split into feedings, either every day (DY) or every other day (ALT). For reference, the steers on DY LO received 0.4% of BW/d and the steers on the DY HI received 0.8% of BW/d of supplement. Supplementation amount was based upon initial BW and was not changed throughout the experiment. Urea was included at 0% (−U) or 1.3% (+U) of the supplement’s dry matter. Steers were housed in individual pens (2.45 × 1.85 m) with feed bunks and water cups in a temperature-controlled room. Each pen had two separate feed bunks, one for supplement and one for hay. Mineral lick blocks, same as those utilized in Exp. 1, were also available in every pen. Supplement was fed at 0700 h immediately followed by hay. Supplement was the same as in Exp. 1. Smooth bromegrass hay (11.5% CP; 64.1% NDF, DM-basis), ground through a 127 mm screen, was fed to attain ad libitum intake. To ensure hay intake was not limited, hay orts were removed and weighed daily. Adjustments to the amount of hay offered were made depending on refusal amount. Periods were 14 d, with 7 d for adaptation. Steers on the ALT treatment received supplement for a total of 7 d during the period (days 2, 4, 6, 8, 10, 12, and 14).

Hay orts during the collection period were subsampled and dried in a forced air oven at 60 °C for 48 h to measure dry matter intake (DMI). No supplement orts were collected due to all animals consumed all supplement offered within 6 h. The same hay that was fed during the trial was also utilized for in situ incubations. Hay was ground through a 2-mm screen using a Wiley mill (No. 4, Thomas Scientific, Swedesboro, New Jersey) and 1.25 g was placed in 5 × 10 cm, 50 µm pore size in situ bags (Ankom Technologies; Macedon, NY). Three in situ bags per time point were placed in a mesh laundry bag with a weight. Bags were inserted in the rumen through cannula at 0700 h then incubated for 4, 8, 12, 24 and 96 h. To determine if there were potential differences in rumen fermentation between days steers received supplement and days they did not, animals on the ALT treatment had two sets of in situ incubations: one on the day of feeding (day 10) and a second on the subsequent non-supplemented day (day 11). However, only one 96 h in situ incubation was conducted and removed on day 14. Animals on the DY treatment had one set of in situ incubations, the same day the ALT animals had their supplemented day collections (day 10). Following all incubation, bags were washed in a standard washing machine with five 1-min agitation, 1-min spin cycles. To account for washout, three unincubated in situ bags were included. Bags were then rinsed with distilled water and frozen. Thawed bags were placed into the Ankom Fiber Analyzer (A2000; Ankom Technologies, Macedon, NY) to determine NDF content.

Rumen fluid was collected at 2, 4, 8, 12, 16, and 24 h post-feeding to analyze rumen ammonia-N. Like the in situ incubations, animals on the ALT treatment had two sets of collections: one on supplemented day (day 12) and not supplemented (day 13). Daily animals had rumen fluid collected on day 12. Approximately 100 mL of rumen fluid was collected via a vacuum hand pump into a 50-mL conical tube and then frozen until analysis.

Ruminal ammonia-N concentration was determined using the alkaline hypochlorite phenol colorometic procedure (Broderick and Kang, 1980) using a Spectramax 250 microplate reader (Molecular Devices, Sunnyvale, CA). Samples were prepared in duplicate.

Rumen pH was measured using intraruminal pH probes (smaXtec Classic Bolus; Graz, Austria). Probes were first calibrated then inserted through the rumen cannula, into the reticulum, prior to the start of the trial and remained through the duration of the trial, a total of 84 d. Readings were collected every 10 min.

Rumination was measured by an ear tag sensor (CowManager; Utrecht, The Netherlands). The ear tag sensor was fit around an RFID tag placed in the animal’s left ear prior to the start of the trial and remained for the entirety of the trial, 84 d. Rumination count was measured every hour.

An additional collection period after the final experimental period in which only hay was fed to all animals was done to provide a reference range for ruminal digestion parameters of the hay in conditions when supplement was not provided. Rumen fluid was collected for ruminal ammonia-N concentration and in situ bags were incubated to determine NDF disappearance rate using the same procedures as stated above. Values from all animals were averaged to get a mean for hay alone.

For the digestion trial to best understand the impacts of frequency, two different data sets were analyzed. One set compared DY to ALT, in which values for each measurement for ALT treatments were averaged across all collection days. Within the ALT treatment, a second analysis which only contained data from ALT steers was conducted to evaluate difference between the day steers were supplemented (ALT-SUP) and the day that they were not (ALT-NSUP).

Digestion and rumen parameters were analyzed using the MIXED procedure of SAS (SAS Ins. Inc, Cary, NC). The model for the DY vs. ALT data set included amount of supplementation, frequency of supplementation, inclusion of urea, and all factorial interactions. The ALT-SUP vs. ALT-NSUP model included amount of supplementation, feeding of supplement, inclusion of urea, and all factorial interactions. Interactions that were not significant (*P* < 0.05) were removed from the models. Time post-feeding was included in both models using repeated measures over time for rumen ammonia-N and pH data. Covariance structure was based on lowest Akaike’s Information Criteria and AR(1) provided the best fit. For DMI, rumination, and in situ NDF disappearance rate, data were analyzed using the MIXED Procedure of SAS (SAS Inst. Inc., Cary, NC). To determine in situ degradation rate, the NLIN Procedure of SAS was used with the Gauss–Newton method to obtain the fractional rate of fermentation per hour (Tedeschi et al., 2009).

## RESULTS

### Exp. 1: Performance Trial

There were no significant (*P* < 0.05) interactions for any of the variables measured. Final body weight did not differ between DY and 3X treatments, nor +U and −U (*P* > 0.56; Table 2). However, final BW was greater (*P* < 0.01) for HI compared to LO steers (319 and 293 kg, respectively). Average daily gain was 0.30 kg greater (*P* < 0.01) for steers receiving a HI amount of supplement than LO. Frequency and urea inclusion did not affect steer ADG (*P* > 0.86). Hay DMI was reduced (*P* < 0.01) by 0.39 kg/d for the steers on the HI treatment compared to LO. Additionally, reduced frequency of supplementation reduced hay (*P* < 0.01) DMI. When averaged across HI and LO treatments over the week, steers receiving 3X supplementation consumed an average of 5.52 kg/d of hay while DY steers consumed 6.06 kg/d. Urea inclusion had no effect on hay DMI (*P* = 0.25).

**Table 2.** Performance of steers fed distillers grains supplement either daily (DY) or 3 times a week (3×), at a high (HI) or low (LO) amount, and with (+U) or without (−U) the inclusion of urea (Exp. 1)

	Treatment									
	Freq^1^		Amt^2^		Urea^3^			P		
	DY	3X	LO	HI	-U	+U	SEM	Freq	Amt	Urea
Initial Body Weight (BW), kg	247	247	247	247	247	247	1.80	0.86	0.72	0.87
Final BW, kg	307	305	293	319	306	306	2.30	0.56	<0.01	0.99
Average Daily Gain, kg	0.72	0.69	0.55	0.85	0.70	0.70	0.01	0.20	<0.01	0.82
Hay Dry Matter Intake, kg/d^4^	6.06	5.52	5.99	5.60	5.89	5.70	0.12	<0.01	<0.01	0.25

DY = daily, 3X = Monday, Wednesday, Friday.

LO = 0.37% of initial body weight, HI = 0.74% of initial body weight.

+U = inclusion of urea at 1.3% of supplement DM, −U = no inclusion of urea.

Averaged across the week.

### Exp. 2: Digestion Trial

#### Supplementation frequency hay intake.

 Both amount and frequency of supplementation impacted hay DMI (*P* < 0.01) of steers during the digestion trial (Table 3). High amount of supplement reduced hay DMI by 0.99 kg/d compared to LO, and ALT reduced hay DMI by 0.47 kg/d compared to DY. Urea inclusion had no significant effect on hay DMI (*P* = 0.21).

**Table 3.** Hay dry matter intake (DMI) of steers fed distillers grains supplement either daily (DY) or alternate days (ALT), at a high (HI) or low (LO) amount, and with (+U) or without (−U) the inclusion of urea during digestion trial(Exp. 2)

	Treatment									
	Freq^1^		Amt^2^		Urea^3^			P		
	DY	ALT	LO	HI	-U	+U	SEM	Freq	Amt	Urea
Hay DMI, kg/d	6.34	5.87	6.60	5.61	5.98	6.22	0.58	<0.01	<0.01	0.21

DY = daily, ALT = every other day.

LO = 0.4% of body weight (BW), HI = 0.8% of BW. Mean BW = 310 kg.

+U = inclusion of urea at 1.3% of supplement DM, −U = no inclusion of urea.

#### In situ NDF disappearance. 

There were no significant three-way interactions (*P* > 0.05) in the model comparing supplementation frequency. Additionally, there were no significant differences (*P* ≤ 0.12) in the washout fraction, nor the potentially digestible fraction. There was an interaction of frequency × amount (*P* = 0.05) for rate of NDF disappearance. The DY-LO had a faster rate (*P* = 0.05) of NDF disappearance (5.22%/h) than DY-HI, ALT-HI, and ALT-LO (4.19%/h, 4.19%/h and 4.20%/h, respectively; Table 4).

**Table 4.** In situ neutral detergent fiber (NDF) disappearance for steers (310 kg) fed hay and distillers grains supplement either daily (DY) or alternate days (ALT), and at a high (HI) or low (LO) amount (Exp. 2)

	Treatment				SEM	P		
	DY		ALT					
	HI^1^	LO	HI	LO		Freq	Amt	Interaction
Washout Fraction	0.25	−0.05	−0.08	0.12	0.12	0.82	0.90	0.51
Potentially Digestible Fraction, %	49.6	51.5	49.1	50.2	0.90	0.12	0.36	0.66
Rate, %/h	4.17^b^	5.22^a^	4.19^b^	4.23^b^	0.24	0.06	0.03	0.05

Within a row, common superscripts indicate no significant difference between means, *P* > 0.05.

LO = 0.4% of body weight, HI = 0.8% of body weight.

Rate of NDF disappearance when fed hay alone was 5.17%/h.

#### Ruminal ammonia-N concentration. 

**Figure 1.**
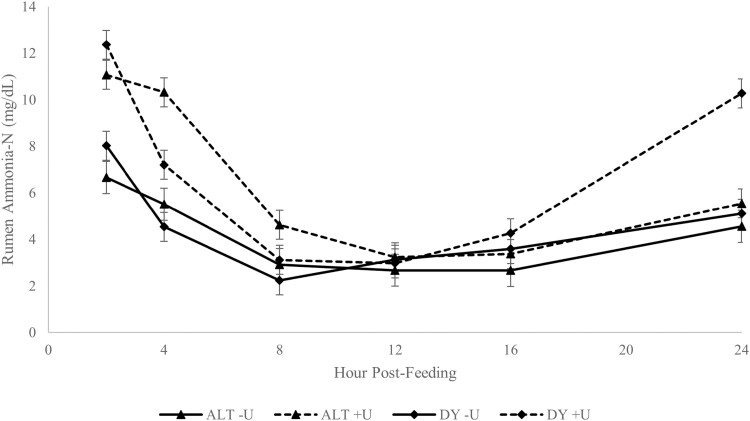
The ammonia-N concentration of ruminal fluid of steers fed distillers grains supplement either every day (DY) or every other day (ALT), and inclusion of urea at 0% (−U) or 1.3% (+U) of the supplement’s dry matter. Frequency × urea × time effect (*P* < 0.01).

There was a significant (*P* < 0.01) frequency × urea × time interaction (Figure 1). There was also a significant interaction of amount × urea (*P* < 0.01). The HI +U had the greatest (*P* < 0.01) average ruminal ammonia concentration, 8.05 mg/dL, whereas HI −U and LO +U did not differ (*P* = 0.97) with an average of 5.00 mg/DL, and LO −U had the lowest (*P* < 0.01), 3.60 mg/dL (Table 5).

**Table 5.** Ruminal ammonia-N concentration for steers fed distillers grains supplement at a high (HI) or low (LO) amount, and with (+U) or without (−U) the inclusion of urea (Exp. 2)

	Treatment					P		
	HI^1^		LO		SEM			
	+U2	−U	+U	−U		Amt	Urea	Interaction
Ammonia-N, mg/dL	8.05^a^	5.00^b^	5.01^b^	3.60^c^	0.325	<0.01	<0.01	<0.01

Within a row, common superscripts indicate no significant difference between means, *P* > 0.05.

By time interaction (*P* < 0.01), data not shown.

LO = 0.4% of body weight (BW), HI = 0.8% of BW. Mean BW = 310 kg.

+U = inclusion of urea at 1.3% of supplement DM, -U = no inclusion of urea.

Ruminal ammonia-N concentration when consuming hay alone was 2.80 mg/dL.

#### Rumen pH.

**Figure 2.**
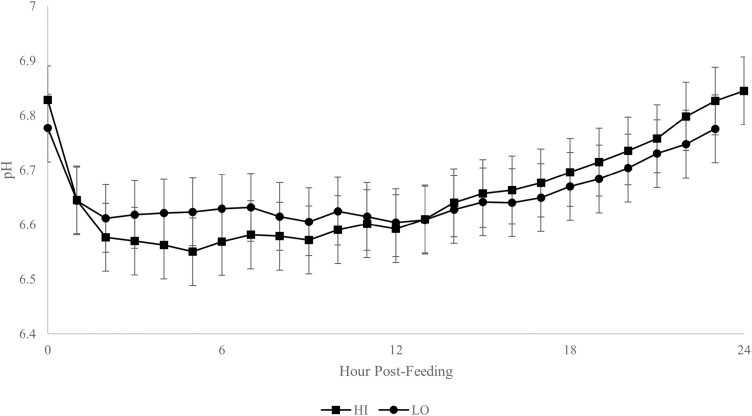
Rumen pH of steers fed distillers grains supplement at a HI (0.8% of body weight) or LO (0.4% of body weight) amount. Mean body weight = 310 kg. Amount × time effect (*P* < 0.01, SEM = 0.062).

There were no significant three-way interactions *(P* > 0.05). There was an interaction (*P* < 0.01) of supplement amount × time on rumen pH. Initially, steers receiving a HI amount had a greater drop in their rumen pH post-feeding than steer receiving a LO amount (Figure 2). However, their pH increased and at approximately 15 h post-feeding was greater (*P* < 0.01) than the steers on the LO amount.

#### Rumination. 

For the supplementation frequency (DY vs. ALT) data set, only the main effect of frequency was significant (*P* < 0.01). Steers that were supplemented daily spent more (*P* < 0.01) minutes per day ruminating than steers that were supplemented every other day (403 vs. 351 min/d; SEM 32.1).

### Day Effect for Alternate Day Supplementation

#### Hay intake.

For the ALT-SUP vs. ALT-NSUP, urea also had no significant effect on hay DMI (*P* = 0.31). There was a significant two-way interaction of day fed × amount (*P* < 0.01). On the day steers were not supplemented ALT-LO and ALT-HI hay DMI did not differ (7.09 kg/d and 6.63 kg/d of hay, respectively). However, the day the ALT treatments were supplemented, ALT-LO consumed more (*P* < 0.01) hay DM than ALT-HI but not as much as on the day they were not supplemented (Table 6).

**Table 6.** Hay dry matter intake (DMI), time spent ruminating, and in situ neutral detergent fiber (NDF) disappearance for steers fed distillers grains supplement on alternative days comparing day fed supplement (ALT-SUP) to day not fed (ALT-NSUP) supplement, and at a high (HI) or low amount (LO) amount of supplement (Exp. 2)

	Treatment					P		
	ALT-SUP		ALT-NSUP					
	HI	LO	HI	LO	SEM	Day Supp^1^	Amt^2^	Interaction
Hay DMI, kg/d	4.38^a^	5.85^b^	6.63^c^	7.09^c^	0.25	<0.01	<0.01	<0.01
Rumination, min/d	297^a^	333^ab^	400^c^	377^bc^	23.8	<0.01	0.70	0.08
In Situ NDF Disappearance								
Washout Fraction	−0.5	−0.2	0.4	0.5	0.7	0.44	0.63	0.91
Potentially Digestible Fraction, %	51.2	49.4	51.8	51.0	1.2	0.31	0.34	0.62
Rate of NDF Digestibility, %/h	3.76^b^	4.72^a^	4.63^ab^	3.75^b^	0.43	0.89	0.92	<0.01

Within a row, common superscripts indicate no significant difference between means, *P* > 0.05.

ALT-SUP = day fed supplement plus hay, ALT-NSUP = day not fed supplement, received hay only.

LO = 0.4% of body weight, HI = 0.8% of body weight. Mean BW = 310 kg.

Rate of NDF disappearance when consuming hay alone was 5.17%.

#### In situ NDF disappearance. 

For day of supplementation within the ALT treatment comparison, there was an interaction of feeding × amount (*P* < 0.01) for rate of NDF disappearance with ALT-SUP LO being greater (*P* < 0.01) than ALT-NSUP HI, ALT-SUP HI and ALT-NSUP LO, which did not differ in NDF disappearance (*P* > 0.05; Table 6). No other interactions or treatment effects were observed for washout fraction, potentially digestible fraction, or rate of NDF disappearance in either model.

#### Ruminal ammonia-N concentration. 

There was a significant interaction of day × amount × urea (*P* < 0.01). Steers on the HI +U treatment on the day they were supplemented, had the greatest ruminal ammonia-N concentration. Steers on treatments without the addition of urea had the lowest (*P* < 0.01) ammonia-N concentrations (Table 7).

**Table 7.** Ruminal ammonia-N concentration for steers consuming hay and fed distillers grains supplement on alternative days comparing day fed supplement (ALT-SUP) to day not fed supplement (ALT-NSUP), at a high (HI) or low amount (LO), and with (+U) or without (−U) the inclusion of urea (Exp. 2)

	Treatment									P			
	ALT-SUP^1^				ALT-NSUP								
	HI^2^		LO		HI		LO						
	+U^3^	−U		−U	+U	−U	+U	−U	SEM	Day Supp	Amt	Urea	Interaction
Ammonia-N, mg/dL	10.56^a^	4.89^c^	5.63^b^	3.58^c^	5.13^bc^	4.49^bc^	4.17^c^	3.78^c^	0.489	<0.01	<0.01	<0.01	<0.01

Within a row, common superscripts indicate no significant difference between means, *P* > 0.05.

Ruminal ammonia-N concentration when fed hay-only was 2.80 mg/dL.

ALT-SUP = day fed supplement plus hay, ALT-NSUP = day not fed supplement, received hay only.

LO = 0.4% of body weight, HI = 0.8% of body weight. Mean BW = 310 kg.

+U = inclusion of urea at 1.3% of supplement DM, −U = no inclusion of urea.

#### Rumen pH.

**Figure 3.**
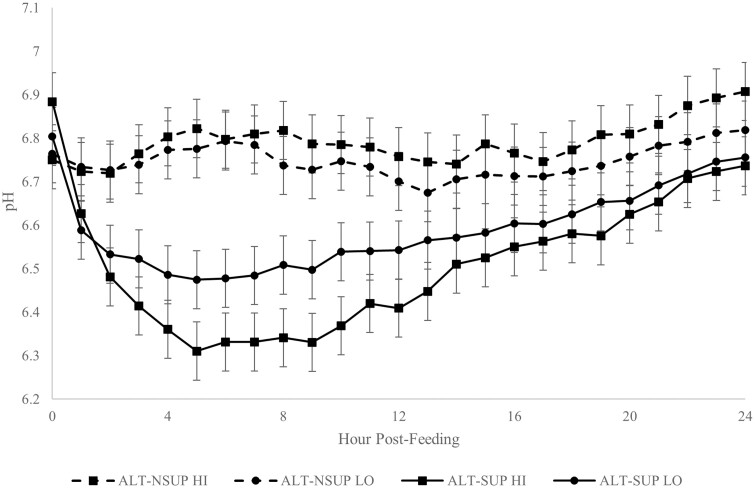
Rumen pH of steers fed distillers grains supplement on alternate days, comparing the day of supplementation (ALT-SUP) to the day of no supplementation (ALT-NSUP), fed at a HI (0.8% of body weight) or LO (0.4% of body weight) amount (Exp. 2). Mean body weight = 310 kg. Supplementation day × amount × time interaction (*P* < 0.01).

There was a significant interaction of feeding × amount × time (*P* < 0.01). On the day they were supplemented, steers had a lower (*P* < 0.01) pH than on the day they were not supplemented. Additionally, while on supplemented days, steers that received a HI amount had a greater (*P* < 0.01) drop in their ruminal pH than steers that received a LO amount. However, on non-supplemented days, there was no significant differences between pH of LO and HI fed steers (Figure 3).

#### Rumination. 

There was a tendency for an interaction (*P* = 0.08) of day supplemented × amount. Steers that received a HI amount ruminated less on the day they were fed supplement (*P* < 0.01; Table 6). Steers that received a LO amount had a tendency (*P* = 0.07) to ruminate less the day they were supplemented vs. the day they were not. However, the magnitude of this change was less for the LO amount of supplement compared to the HI. There was a main effect of day fed for the ALT-SUP vs. ALT-NSUP data (*P* < 0.01). Animals ruminated 75.54 more minutes per day on the day they did not receive supplemented than the day they did (Table 6).

## DISCUSSION

Growing steer ADG was only impacted by increasing the amount of DDGS supplement. Other supplementation studies have reported increases in gain with increasing amount of DDGS supplementation (Loy et al., 2008). An increased amount of supplementation also led to a reduction in hay DMI. Again, this effect has been observed as there is a substitution of forage intake with increasing supplement intake. Horn and McCollum (1987) observed a forage replacement effect when the supplementation rate was greater than 0.50% of BW daily. Furthermore, Loy et al. (2008) reported a 0.78 kg/d difference in hay DMI between steers supplemented with DDGS at 0.21% or 0.81% of BW. In the current study, the steers on the DY LO treatment received approximately 0.4% of their BW while steers on DY HI received 0.8% of their BW. Supplementation frequency also reduced hay DMI, despite receiving the same amount of supplementation weekly, ALT steers did not consume as much hay as those on the DY treatment. This effect was also observed by Loy et al. (2007, 2008) and Drewnoski et al. (2011, 2012). Additionally, the day in which ALT supplemented cattle received supplement and the amount impacted their hay consumption. Days in which ALT treatments did not receive supplement, they consumed more hay than the day they were supplemented, with both the HI and LO steers consuming the same amount on non-supplemented days. However, days the ALT treatment steers were supplemented, those receiving a HI amount consumed 1.47 kg/d less than steers receiving a LO amount. Loy et al., (2007) also observed a decrease in hay intake on the days in which alternative supplemented heifers received their supplement, reporting an 11% decrease in hay DMI. Rumination data followed hay intake, as consuming more hay led to an increase in total rumination time. Previous research has shown that due to their smaller particle size concentrates, including distillers, do not stimulate rumination (Sudweeks et al., 1975; Penner et al., 2009); thus, amount of time spent ruminating is driven by forage NDF intake.

Forage intake in ruminants can be influenced by two factors, physical/feed factors, or physiological/animal factors (Van Soest, 1994). In the case of physical factors, fill of the rumen limits intake, while in physiological factors, metabolic feedback is the limiter. Since forage intake is correlated with NDF content of the forage, decreasing the NDF digestibility in the rumen could lead to decreased intake. However, in the present study there was no significant difference between DY and ALT in situ NDF disappearance rate. This suggests that changes in rumen fill are not the reason for the reduced intake. When a mix of soyhulls and corn gluten feed was supplemented every other day, hay intake on the day steers that did not receive supplement was less than the hay only control, suggesting that there is some carry-over effect of supplementation from the previous day (Drewnoski and Poore, 2012). In their study, the total VFA concentrations of the ALT steers was elevated above the hay only control for 32 h after supplementation, suggesting that the reduced hay intake may be due to metabolic feedback.

Rumen pH is often cited as a factor that impairs fiber digestion as fibrolytic bacteria are most sensitive to a pH below 6.2 (Grant and Mertens, 1992; Russell and Wilson, 1996). Some supplements when fed infrequently have reduced rumen pH below 6.2 but these supplements were high in NSC and often did not meet RDP requirements (Caton and Dhuyvetter, 1997). However, since DDGS contains little NSC, no treatment reduced rumen pH below 6.2. Additionally, frequency of supplementation had no significant impact on ruminal pH. Although there was an interaction between supplementation feeding by amount for ALT animals, even on supplementation days rumen pH was greater than 6.2. Although the pH for the ALT-SUP HI animals never dropped below 6.2, there was a reduction in in situ NDF digestibility for this treatment compared to the ALT-SUP LO. It is possible the time near a pH of 6.2 may have reduced in situ NDF digestibility, but it was not enough to impact on animal performance.

Unlike previous studies with DDGS, infrequent supplementation with DDGS did not reduce steer ADG. Loy et al. (2008) observed a 10% reduction in ADG when DDG was fed 3×/weekly compared to daily. Likewise, Stalker et al. (2009) reported a 10% decrease in ADG from 6×/weekly DDGS supplementation to 3×/weekly. The results of the current study agree with those of Drewnoski et al. (2011), which reported no difference in ADG of steers supplemented 7×, 3×, or 2×/weekly with a corn gluten feed and soy hulls blend. However, the differences in the supplement types may have resulted in the difference in animal performance. The supplement in both Loy et al. (2008) and Stalker et al. (2009) contains a greater amount of fat (ether extract = 10.2%) than the current experiments as well as less RDP than the supplement utilized by Drewnoski et al. (2011). Both dietary fat and RDP can impact rumen fibrolytic bacteria, reducing forage digestion and thus, animal performance.

However, inclusion of urea, a RDP source, had no impact on animal performance or hay intake. Satter and Slyter (1974) reported that fibrolytic bacteria growth is inhibited when rumen ammonia concentration was below 2 mg/dL. While in the digestion trial there was a significant amount x urea x time interaction, none of the treatments had rumen ammonia-N concentrations drop below 2 mg/dL. Additionally, urea had no significant effect on in situ NDF disappearance, also suggesting that the RAN pool was not limiting for fiber digestion. Our hypothesis that infrequent supplementation resulted in asynchrony between rumen available energy and rumen available nitrogen, leading to a reduction in forage digestibility was not support by data in either the performance or digestion trial. It is important to note that hay in the digestion trail likely did not result in a RAN deficiency, given that it was 11.5% CP. The hay in the performance trial was 6.8% CP and thus more likely resulted in a RAN deficiency. Yet, there was still no improvement in animal performance when urea was included in the supplement nor a difference between daily and infrequent supplementation.

One key difference between the DDGS supplement fed in Loy et al. (2007, 2008) and Stalker et al. (2009) and these studies was the fat content. Distillers grains processing methods have changed since the early 2000s with processing plants extracting more of the fat. Currently, fat is centrifuged from the solubles stream. The fat removed through this process is more reactive in the rumen than the remaining fat in DDGS, which is held in the corn germ and likely bypasses. Since this centrifugation process was not commonly used during the time of Loy et al. (2007, 2008) and Stalker et al. (2009), the fat in the DDGS supplement in these studies was more reactive in the rumen environment. The ether extract content of DDGS in the previous studies was approximately 10–11%, whereas the ether extract of the DDGS utilized in the current studies was ~5%. The supplement in Drewnoski et al. (2011, 2012) also contained little EE, the value was not reported for the supplement itself, but CGF has ~3.5 % and SH ~2.2% ether extract (NASEM, 2016). Fat can reduce fiber digestion in the rumen as it is toxic to fibrolytic bacteria and can reduce the time for bacterial attachment to forage particles (Jenkins, 1993). In Loy et al. (2008), the total fat content of the diet was 5.2% and in Stalker et al. (2009) feeding their supplement 3×/weekly resulted in an additional 5.4% of fat to the diet. Feeding supplement less frequently would require a larger amount to be fed per feeding, resulting in a greater percentage of the diet as fat on the day of supplementation. It is recommended that dietary fat content does not exceed 5% in forage-based diets, but in both Loy et al. (2008) and Stalker et al. (2009), dietary fat content exceeded that for animals receiving infrequent supplementation. This could have had negative effects on fiber digestion in the rumen, reducing forage utilization and subsequent animal performance. Interestingly though, a digestion study done by Loy et al. (2007) with DDGS containing 9.67% ether extract saw no reduction in in situ NDF disappearance rate when supplement was fed infrequently compared to daily. Likewise, there was no significant difference between DY and ALT in situ NDF disappearance rate in the current digestion trial. However, Stalker et al. (2009) observed a linear decrease in total tract NDF digestibility as supplementation frequency decreased. Drewnoski and Poore (2012) also used total tract digestibility but did not see a difference in the potentially digestible NDF digestibility between frequency of supplementation. Furthermore, none of the previously mentioned studies with DDGS measured passage rate. Drewnoski and Poore (2012) did measure total tract passage rate through a rare earth marker tagged to the SH in the supplement but did not report a difference in total tract passage rate between frequent and infrequently supplemented animals. However, this was also total tract passage rate, not just rumen passage rate. Rumen digestibility is impacted by both digestibility rate and passage rate. These factors have an inverse relationship, as increasing passage rate will decrease digestibility rate. Conversely, decreasing ruminal passage rate allows for an increase in digestibility rate but can reduce animal performance. Increasing the amount of supplementation would increase rumen passage rate but decrease digestibility. However, without measuring rumen passage rate, it is difficult to determine if supplementation frequency impacts rumen digestibility by altering passage rate.

## IMPLICATIONS

The results of these studies suggest that a DDGS supplement with a lower fat content can be fed infrequently to growing steers on a high forage diet with no reduction in performance. Additionally, decreasing supplementation frequency can reduce hay dry matter intake. Including urea had no impact on animal performance or hay DMI nor any ruminal digestion parameters, suggesting that there is no deficiency in the RAN pool leading to a reduction in forage utilization.
